# It Has Not Been a Good Day: Managing Difficult Behaviors of Relatives With Dementia

**DOI:** 10.1093/geroni/igaa057.2192

**Published:** 2020-12-16

**Authors:** Karen Roberto, Brandy Renee McCann, Tina Savla, Emily Hoyt, Rosemary Blieszner, Aubrey Knight

**Affiliations:** 1 Virginia Tech, Blacksburg, Virginia, United States; 2 Virginia Polytechnic Institute and State University, Blacksburg, Virginia, United States; 3 Virginia Tech Carilion School of Medicine, Roanoke, Virginia, United States

## Abstract

Most family caregivers provide appropriate care and a supportive environment for their older relatives with dementia (PwD), yet the stress and strain associated with caregiving can trigger potentially harmful responses. Although much has been written about dealing with memory problems, researchers know less about how caregivers cope with difficult behaviors such as hallucinations, violent outbursts, or refusing food, medicine, or bathing. Interviews with 30 relatives providing care to community-dwelling PwD in rural Virginia revealed that caregivers typically used four behavior management strategies: reasoning with PwD; redirecting PwD’s attention; forceful actions, such as shouting at PwD; and withdrawing from interactions. Forceful management strategies and withdrawing from interactions were usually employed after reasoning and redirection failed to elicit desired behavior. Understanding whether caregivers’ expectations of PwD’s capacities are realistic, and why and when caregivers use various behavior management strategies, can help service providers develop appropriate educational interventions for frustrated caregivers.

